# Difficulty with *Gordonia bronchialis* identification by Microflex mass spectrometer in a pacemaker‐induced endocarditis

**DOI:** 10.1099/jmmcr.0.003681

**Published:** 2014-09-01

**Authors:** Marie Titécat, Caroline Loïez, René J. Courcol, Frédéric Wallet

**Affiliations:** 1 Institute of Microbiology, University Hospital Centre, Lille, France; 2 University of Lille Nord de France, Lille, France

**Keywords:** 16S rRNA, endocarditis, *Gordonia bronchialis*, MALDI‐TOF MS, pacemaker, *rpo*B sequencing

## Abstract

**Introduction:**

This report describes the first case, to the best of our knowledge, of pacemaker‐induced endocarditis due to *Gordonia bronchialis*.

**Presentation:**

Pacemaker‐induced endocarditis due to *G. bronchialis* infection was determined in a 92‐year old man. This Gram‐positive bacillus failed to be identified by matrix‐assisted laser desorption/ionization time‐of‐flight mass spectrometry technology, whereas the taxon was indexed in the database. 16S rRNA and *rpoB* gene sequencing were required to determine the correct strain identity.

**Conclusion:**

Infections caused by *G. bronchialis* remain a rare phenomenon affecting immunocompromised patients and/or medical device carriers. Molecular tools may be necessary to ensure accurate identification.

## Case report

A 92‐year old man who underwent pacemaker implantation 20 years ago was referred to our institution for management of a local pocket infection. The patient’s medical history included high blood pressure, atrial fibrillation and pulmonary embolism. A first management consisting of local surgical intervention and antimicrobial therapy (pristinamycin‐oxacillin) had been performed the previous month in another hospital. On admission, physical examination revealed blood pressure at 120/75 mmHg with cardiac frequency at 78 beats min^−1^ and a grade III New York Heart Association dyspnoea. The patient had no peripheral stigmata of endocarditis. The leukocyte count was 6×10^3^ µl^−1^ and his C‐reactive protein level was 3 mg l^−1^ (normal value <10 mg l^−1^). As no fever was noted, no blood culture was performed. Transthoracic echocardiography showed a vegetation (12×5 mm) on the ventricular lead in the right auricle. As the local infective process did not heal, and according to the echocardiography data, it was decided to rapidly remove the whole pacemaker (generator and leads) without further bacteriological investigations (blood culture). Different parts of the device were plated onto chocolate and 5 % sheep blood agar for aerobic culture and inoculated into broths for anaerobic culture as described previously (Klug *et al*., 2004). After 2 days of incubation, aerobic cultures from all samples grew on blood agar with small dry greyish colonies with a wrinkled aspect in pure culture. The colonies were non‐haemolytic, did not produce any aerial hyphae and showed a yellow orange pigmentation after 4 days of incubation. This beaded Gram‐positive bacillus was catalase positive and oxidase negative.

Matrix‐assisted laser desorption/ionization time‐of‐flight mass spectrometry (MALDI‐TOF MS) identification was performed on a 96‐target plate (Bruker Daltonics) with a Microflex mass spectrometer (Bruker Daltonics) using FlexControl software (version 3.0). The spectrum was imported and compared with the database by the BioTyper software (version 2.0; Bruker). The BioTyper database contains spectra of approximately 4613 species and is regularly updated by the manufacturer. Theoretically, an accurate identification score to species level is given by a score value ≥2.0 according Bruker recommended cut‐off values. The first result was not conclusive with a score of <1.5. The strain was retested after a protein extraction step, as described previously (Bizzini *et al*., 2010; Khot *et al*., 2012). *Arthrobacter castelii* (score = 1.967) was considered as a reliable identification to species level according to the recently score proposed by Alatoom *et al*. (2012). However, the discrepancy between colonial aspects (i.e. smooth blade yellow colonies for *Arthrobacter* (Heyrman *et al*., 2005) and wrinkled yellow orange colonies for our isolate) led us to perform 16S rRNA gene bacterial sequencing using the primers described by Gauduchon *et al.* (2003). The 471 bp fragment obtained and compared with GenBank sequences using the blast algorithm (http://www.ncbi.nlm.nih.gov/BLAST) showed 99 % identity with *Gordonia bronchialis* strain X0217 (GenBank accession no. HQ316182) and *Gordonia terrae* strain KNUC2111 (GenBank accession no. JN382216).

These results were confirmed by bioinformatic bacterial identification analysis (http://pbil.univ-lyon1.fr/bibi/query.php) with 19 choices for *G. bronchialis* and one for *G. terrae*. Sequencing of the *rpoB* gene was then performed with primers C2700F and C3130R described by Khamis *et al*. (2004). The 413 bp fragment showed 99 % nucleotide similarity to *G. bronchialis* strain DSM 43247 (GenBank accession no. CP001802). Antimicrobial susceptibility testing performed by the disc diffusion method on Mueller–Hinton agar with the addition of 5 % blood and interpreted according to EUCAST (2014) criteria showed susceptibility to amoxicillin, piperacillin, cefotaxime, imipenem, aminoglycosides, trimethoprim‐sulfamethoxazole, fluoroquinolones, vancomycin and linezolid and resistance to macrolides and tigecyclin. According to these data, the first line of antimicrobial treatment by intravenous piperacillin‐tazobactam and daptomycin was switched for amoxicillin *per os*. The clinical outcome was favourable after 6 weeks of treatment.

## Discussion

The genus *Gordonia*, initially named ‘*Gordona*’ (Tsukamura, 1971), belongs to the suborder *Corynebacteriaceae*. This taxon became a well‐defined genus within the order *Actinomycetales* (Stackebrandt *et al*., 1997). *Gordonia* spp. are aerobic bacteria widely distributed in the environment (soil and water) (Tsukamura, 1971). Based on 10 years of Medline research using *Gordonia* and endocarditis, this is the first reported case, to the best of our knowledge, of endocarditis due to *G. bronchialis* in a pacemaker holder. Two other cases of native valve endocarditis were reported due to two other species: *Gordonia polyisoprenivorans* and *Gordonia* sp. most closely resembling *Gordonia sputi*, in two immunocompromised patients (Lesens *et al*., 2000; Verma *et al*., 2006). *G. bronchialis* was clearly involved in seven relevant documented adult infections, described in Table 1 (Sng *et al*., 2004; Werno *et al*., 2005; Johnson *et al*., 2011; Siddiqui *et al*., 2012; Wright *et al*., 2012). This bacterium is an opportunistic pathogen inducing a weak immune response from the host. The genus *Gordonia* is able to form biofilm by producing gordonan, an acidic cell aggregation‐inducing polysaccharide, and consequently is responsible for persistent infections (Kondo *et al*., 2000). In our observation, *G. bronchialis* was involved in a pacemaker infection in a patient with severe underlying disease. No recommendation for antimicrobial susceptibility testing has been approved. This microorganism appears to be highly susceptible to most antibiotics, as shown by the different regimens applied successfully in the different case reports (Table 1). However, in *G. bronchialis* infections, prolonged antimicrobial treatment is required, and remission may be attested after a prolonged follow‐up period.

**Table 1.** Reported infections attributable to *G. bronchialis*

Reference	Type of infection	No. cases/sex (F/M)/age (years)	Underlying condition	Fever ≥38 °C	Clinical device	Successive treatments	Outcome
Sng *et al*. (2004)	Bacteraemia and sequestrated lung	1F/58	Mellitus, asthma	Yes	No	Surgical drainage, 3 months vancomycin+ceftriaxone i.v., 6 weeks AMC p.o.	Cure
Werno *et al*. (2005)	Recurrent breast abscess	1F/43	Pituitary adenoma	No data	No	3 Days penicillin+flucloxacillin i.v., 3 days AMC+metronidazole p.o., 12 days doxycycline+clindamycin p.o. Surgical drainage, and 5 months doxycycline p.o.	Recurrence
Johnson *et al*. (2011)	Bacteraemia and pleural infection	1F/52	Hodgkin lymphoma, splenectomy, breast cancer	No	Pleural catheter	4 Days vancomycin+ceftazidime i.v., TMP/SMX+imipenem i.v., TMP/SMX p.o. switched by ciprofloxacin+minocycline p.o. for 3 months	Cure
Siddiqui *et al*. (2012)	Osteomyelitis	1F/22	Knee surgery	No	Bioresorbable screw	Screw removal, 2 weeks vancomycin i.v., 4 weeks ciprofloxacin p.o.	Cure
Wright *et al*. (2012)	Sternal wound infection	3M/56–80	Mellitus for 2 patients	No	Coronary‐artery bypass (grafts)	41–77 Days (mean 60) imipenem i.v. (for the 3 patients) debridement 8 weeks moxifloxacin+linezolid+minocycline p.o. (for 1 patient)	Cure
This case	Pacemaker ‐induced endocarditis	1M/92	High blood pressure, Pulmonary embolism	No	Pacemaker	Pacemaker removal, 6 days PTZ+daptomycin i.v., 6 weeks amoxicillin i.v.	Cure

F, female; M, male; AMC, amoxicillin‐clavulanic acid; TMP/SMX^;^ trimethoprim‐sulfamethoxazole; PTZ, piperacillin‐tazobactam; p.o., per os; i.v., intravenous.

More generally, this case underlines the inability of the available identification systems to accurately identify *Gordonia* spp. This difficulty has largely been commented on in previous articles using the commercial API Coryne system (bioMérieux), which were unable to identify members of the genus *Gordonia* as it was not indexed in the system’s library (Sng *et al*., 2004; Werno *et al*., 2005; Verma *et al*., 2006). Even with protein extraction, MALDI‐TOF MS failed to give the right identification. This might be due to the weak representation of this species in the Bruker library (one spectrum), explaining the misidentification of our isolate as *A. castelii* (Seng *et al.*, 2009). This technology emerged in recent years as a fast and reliable technique aimed at replacing conventional phenotypic identification methods (Bizzini *et al*., 2010; Khot *et al*., 2012). However, the present case leads us to remain critical towards MS results for uncommon Gram‐positive bacillus identification, even with good identification scores. Once again (Sng *et al*., 2004; Werno *et al*., 2005; Johnson *et al*., 2011; Siddiqui *et al*., 2012; Wright *et al*., 2012), molecular sequencing was required to determine the strain identity. Although 16S rRNA gene sequencing is widely used, the *rpoB* gene has been described as a reliable molecular marker for *Corynebacterium* spp. identification (Khamis *et al*., 2004). In this case, both targets gave the same result.

In conclusion, infections caused by *G. bronchialis* remain a rare phenomenon affecting immunocompromised patients and/or medical device carriers. Molecular tools may be necessary to ensure accurate identification of this Gram‐positive bacterium until there is a wider representation of this species in the Bruker library.

## Abbreviations

MALDI‐TOF MS: matrix‐assisted laser desorption/ionization time‐of‐flight mass spectrometry.
